# Manufacture of Anti=Diphtheritic Serum

**Published:** 1898-12

**Authors:** 


### Manufacture of Anti-Diphtheritic Serum.

structive account of his visit to and inspection of the great laboratories of Parke, Davis & Co., Detroit. We abstract, for the information of our readers, the following remarks by Dr. Powell, on the methods adopted by this firm in manufacturing diphtheritic serum, a now staple commodity, which no physician can afford to do without:

"Any physician who will take the time and trouble to thoroughly inspect the methods followed by Parke, Davis & Company in the manufacture of anti-diphtheritic serum cannot but be convinced that whatever virtue resides in the antitoxic serum therapy may be confidently expected to make itself manifest from the employment of their product. The perfect health of the horses, assured by a veterinary surgeon who has had many years' experience in an official capacity as State Veterinarian; antiseptic precautions at every step of the process; the rigid testing upon guinea pigs to prove, in the first place, the strength of the diphtheria toxin, and in the second place the antitoxic strength of the serum; the placing of the product in sterilized glass bulbs, which are immediately sealed so that all atmospheric contamination is absolutely excluded; the dating of each package of antitoxin before it leaves the shipping department, with the guarantee extended to all dealers that the serum is what it claims to be, and that any deterioration through age will be made good by fresh supplies,—all these safeguards, especially when we remember that this work is in charge of a bacteriologist of national repute, Dr. Chas. T. McClintock, with whom are associated such expert pharmacologists as Dr. Houghton, and a small army of trained assistants, certainly leave no room to doubt that whatever bacteriological skill can do has been done to make Parke, Davis & Company's antitoxin an ideal product."

A copy of Dr. Powell's write-up will be sent free on application.
Louis. October, 1898),Dr. C. H. Powell gives an interesting and in-
In *The North American Journal of Diagnosis and Practice* (St.

